# Evaluating Functional Annotations of Enzymes Using the Gene Ontology

**DOI:** 10.1007/978-1-4939-3743-1_9

**Published:** 2017

**Authors:** Gemma L. Holliday, Rebecca Davidson, Eyal Akiva, Patricia C. Babbitt

**Keywords:** Catalytic function, Enzyme, Misannotation, Evidence of function

## Abstract

The Gene Ontology (GO) (Ashburner et al., Nat Genet 25(1):25–29, 2000) is a powerful tool in the informatics arsenal of methods for evaluating annotations in a protein dataset. From identifying the nearest well annotated homologue of a protein of interest to predicting where misannotation has occurred to knowing how confident you can be in the annotations assigned to those proteins is critical. In this chapter we explore what makes an enzyme unique and how we can use GO to infer aspects of protein function based on sequence similarity. These can range from identification of misannotation or other errors in a predicted function to accurate function prediction for an enzyme of entirely unknown function. Although GO annotation applies to any gene products, we focus here a describing our approach for hierarchical classification of enzymes in the Structure-Function Linkage Database (SFLD) (Akiva et al., Nucleic Acids Res 42(Database issue):D521–530, 2014) as a guide for informed utilisation of annotation transfer based on GO terms.

## 1 Introduction

Enzymes are the biological toolkit that organisms use to perform the chemistry of life, and the Gene Ontology (GO) [1] represents a detailed vocabulary of annotations that captures many of the functional nuances of these proteins. However, the relative lack of experimentally validated annotations means that the vast majority of functional annotations are electronically transferred, which can lead to erroneous assumptions and missannotations. Thus, it is important to be able to critically examine functional annotations. This chapter describes some of the key concepts that are unique for applying GO-assisted annotation to enzymes. In particular we introduce several techniques to assess their functional annotation within the framework of evolutionarily related proteins (superfamilies).

### 1.1 Enzyme Nomenclature and How It Is Used in GO

At its very simplest, an enzyme is a protein that can perform at least one overall chemical transformation (the function of the enzyme). The overall chemical transformation is often described by the Enzyme Commission (EC) Number [2–4] (and *see* Chap. 19 [5]). The EC Number takes the form A.B.C.D, where each position in the code is a number. The first number (which ranges from 1 to 6) describes the general class of enzyme, the second two numbers (which both range from 1 to 99) describe the chemical changes occurring in more detail (the exact meaning of the numbers depends on the specific class of enzyme you are looking at) and the final number (formally ranging from 1 to 999) essentially describes the substrate specificity. The EC number has many limitations, not least the fact that it doesn’t describe the mechanism (the manner in which the enzyme performs its overall reaction) and often contains no information on cofactors, regulators, etc. Nor is it structurally contextual [6] in that similarity in EC number does not necessarily infer similarity in sequence or structure, making it sometimes risky to use for annotation transfer, especially among remote homologous proteins. However, it does do exactly what it says on the tin: it defines the overall chemical transformation. This makes it an important and powerful tool for many applications that require a description of enzyme chemistry.

**Fig. 1:**
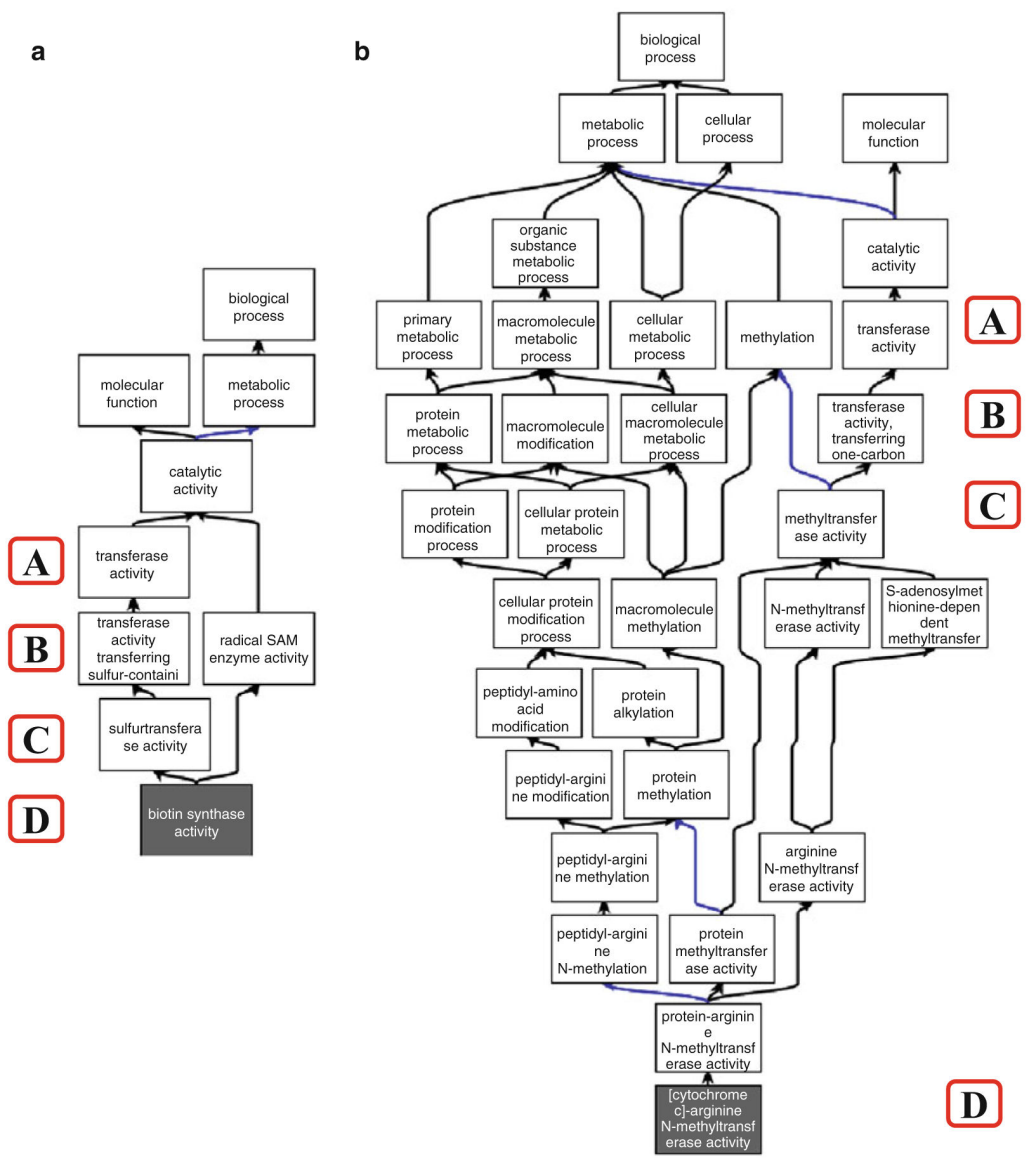
Example of the GO hierarchy (taken from the ancestor chart of the QuickGo website (http://www.ebi.ac.uk/QuickGO/) showing the relative complexity of the GO hierarchy for two distinct EC numbers). (**a**) Shows the GO hierarchy for biotin synthase, EC 2.8.1.6; (**b** ) shows the GO hierarchy for [cytochrome c]-arginine *N* -methyltransferase, EC 2.1.1.24. The *colours* of the *arrows* in the ontology are denoted by the key in the centre of the figure. *Black connections* between terms represent an is_a relationship, *blue connections* represent a part_of relationship. The *A*, *B*, *C* and *D* in *red boxes* denote the four levels of the EC nomenclature

The Molecular Function Ontology (MFO) in GO contains the full definition of around 70 % of all currently available EC numbers. Theoretically, the MFO would contain all EC numbers available. However, due to many EC numbers not currently being assigned to a specific protein identifier within UniProtKB, the coverage is lower than might be expected. Another important difference between the EC hierarchy and the GO hierarchy is that the latter is often much more complex than the simple four steps found in the EC hierarchy. For example, the biotin synthase (EC 2.8.1.6) hierarchy is relatively simple and follows the four step nomenclature, while the GO hierarchy for [cytochrome c]-arginine N-methyltransferase (EC 2.1.1.124) is much more complex (*see* Fig. 1).

Formally, MFO terms describe the activities that occur at the molecular level; this includes the “catalytic activity” of enzymes or “binding activity”. It is important to remember that EC numbers and MFO terms represent activities and not the entities (molecules, proteins or complexes) that perform them. Further, they do not specify where, when or in what context the action takes place. This is usually handled by the Cellular Component Ontology. The final ontology in GO, the Biological Process Ontology (BPO), provides terms to describe a series of events that are accomplished by one or more organised assemblies of molecular functions. Each MFO term describes a unique single function that means the same thing regardless of the evolutionary origin of the entity annotated with that term. Although the BPO describes a collection of activities, some BPO terms can be related to their counterparts in the MFO, e.g. GO:0009102 (biotin bio-synthetic process) could be considered to be subsumed with the MFOtermGO:0004076(biotinsynthaseactivity)asGO:0009102 includes the activity GO:0004076, i.e. in such cases, the terms are interchangeable for the purpose of evaluation of a protein’s annotation. Please *see* Chap. 2 [7] for a more in-depth discussion of the differences between BPO and MFO.

**Fig. 2:**
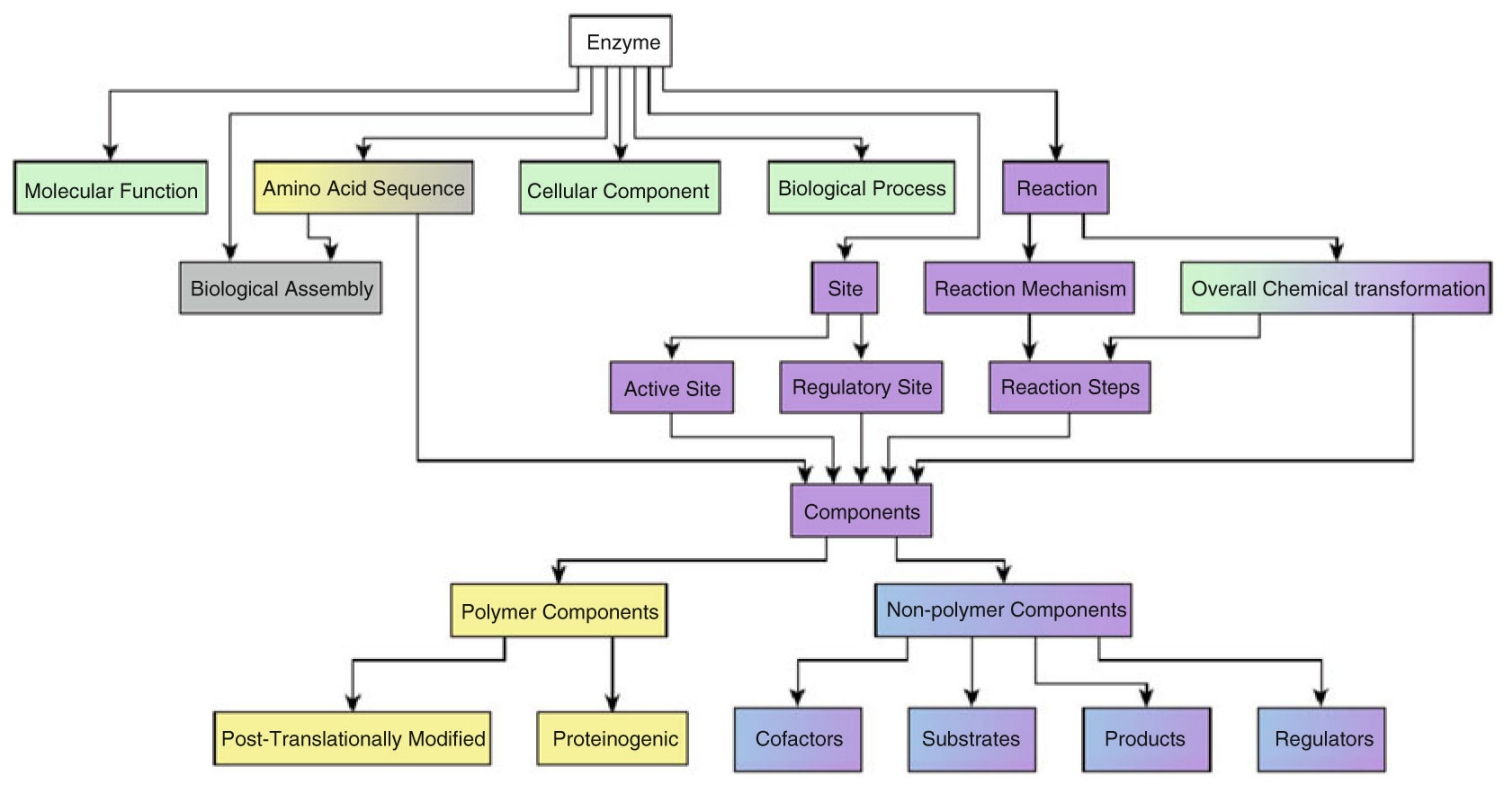
Hierarchical view of enzyme features. The GO ontologies which describe proteins and their features are highlighted in *light green*. Other ontologies available in OBO and BioPortal are shown in the following colours: *light yellow* represents the Amino Acid Ontology, *purple* represents the Enzyme Mechanism Ontology, *blue* represents the ChEBI ontology and *grey* represents the Protein Ontology. *See* also Chap. 5 [10]. The terms immediately beneath the parent term are those terms that are covered by ontologies, and required for a protein to be considered an enzyme

As a protein, an enzyme has many features that can be described and used to define the enzyme’s function, from the primary amino acid sequence to the enzyme’s quaternary structure (biological assembly), the chemistry that is catalysed, to the localisation of the enzyme. Features can also denote the presence (or absence) of active site residues to confirm (or deny) a predicted function, such as EC class, using the compositional makeup of a protein amino acid sequences [8, 9]. Nevertheless, for the many proteins of unknown function deposited in genome projects, prediction of the molecular, biological, and cellular functions remains a daunting challenge. Figure 2 provides a view of enzyme-specific features along with the GO ontologies that can also be used to describe them. Because it captures these features through a systematic and hierarchical classification system, GO is heavily used as a standard for evaluation of function prediction methods. For example, a regular competition, the Critical Assessment of Functional Annotation (CAFA) has brought many in the function prediction community together to evaluate automated protein function prediction algorithms in assigning GO terms to protein sequences [11]. Please *see* Chap. 10 [12] for a more detailed discussion of CAFA.

### 1.2 Why Annotate Enzymes with the Gene Ontology?

Although there are many different features and methods that can (and are) used to predict the function of a protein, there are several advantages to using GO as a broadly applied standard. Firstly, GO has good coverage of known and predicted functions so that nearly all proteins in GO will have at least one associated annotation. Secondly, annotations associated with a protein are accompanied by an evidence code, along with the information describing that evidence source. Within the SFLD [13] each annotation has an associated confidence level which is linked to both the evidence code, source of the evidence (including the type of experiment) and the curator’s experience. For example, experimental evidence for an annotation is considered as having high confidence whereas predictions generated by computational methods are considered of lower confidence (Chap. 3 [14]). In general there are three types of evidence for the assignment of a GO term to a protein:

1. Fully manually curated: These proteins will usually have an associated experimental evidence that has been identified by human curators and who have added relevant evidence codes. For the purposes of the SFLD and this chapter, these are considered high confidence and will have a greater weight than any other annotation confidence level.
2. Computational with some curator input: These are computationally based annotations that have been propagated through curator derived rules, and are generally considered to be of medium confidence by the SFLD. Due to the huge proportion of sequences in large public databases now available, over 98 % of GO annotations are inferred computationally [15].
3. Computational with no curator input: These annotations that have been computationally inferred from information without any curator input into the inference rules and are considered to be of the lowest confidence by the SFLD.

All computationally derived annotations rely upon prior knowledge, and so if the rule is not sufficiently detailed, it can still lead to the propagation of annotation errors (*see* Misannotation Section 1.4).

Assigning confidence to annotations is highly subjective [16], however, as one person may consider high-throughput screening, which more frequently is used to predict protein-binding or sub-cellular locations rather than EC number, of low confidence. This is because such experiments often have a relatively high number of false positives that can generate bias in the analysis. However, depending on what your research questions are, you may consider such data of high confidence. It all depends on what field you are in and what your needs are. Generally speaking, the more reproducible the experiment(s), the higher confidence you can have in their results. Thus, even low-to-medium confident annotations (from Table 1) may lead to a high-confidence annotation.

For example the GO Reference Code GO_REF:0000003 provides automatic GO annotations based on the mapping of EC numbers to MFO terms, so although annotated as IEA, these annotations can be considered of higher confidence [18]. Some examples of high-, medium- and low-confidence annotations are shown in Table 1, along with reference to the approach used in SwissProt and the SFLD to describe their reliability.

### 1.3 Annotation Transfer Under the Superfamily Model

We define here an enzyme (or protein) superfamily as the largest grouping of enzymes for which a common ancestry can be identified. Superfamilies can be defined in many different ways, and every resource that utilises them in the bioinformatics community has probably used a slightly different interpretation and method to collate their data. However, they can be broadly classified as structure- based, in which the three-dimensional structures of all available proteins in a superfamily have been aligned and confirmed as homologous, or sequence based, where the sequences have been used rather than structures. Many resources use a combination of approaches. Examples of superfamily based resources include CATH [19], Gene3D [20], SCOP and SUPERFAMILY [21], which are primarily structure based, and Pfam [22], PANTHER [23] and TIGRFAMs [24], which are primarily sequence based. A third definition of a superfamily includes a mechanistic component, i.e. a set of sequences must not only be homologous, but there must be some level of conserved chemical capability within the set, e.g. catalytic residues, cofactors, substrate and/or product substructures or mechanistic steps. An example of such a resource is the SFLD and we will focus on this resource with respect to evaluating GO annotations for enzymes that are members of a defined superfamily.

The SFLD (http://sfld.rbvi.ucsf.edu/) is a manually curated classification resource describing structure-function relationships for functionally diverse enzyme superfamilies [25]. Members of such superfamilies are diverse in their overall reactions yet share a common ancestor and some conserved active site features associated with conserved functional attributes such as a partial reaction or molecular subgraph that all substrates or products may have in common. Thus, despite their different functions, members of these superfamilies often “look alike” which can make them particularly prone to misannotation. To address this complexity and enable reliable transfer of functional features to unknowns only for those members for which we have sufficient functional information, we subdivide superfamily members into subgroups using sequence information (and where available, structural information), and lastly into families, defined as sets of enzymes known to catalyse the same reaction using the same mechanistic strategy and catalytic machinery. At each level of the hierarchy, there are conserved chemical capabilities, which include one or more of the conserved key residues that are responsible for the catalysed function; the small molecule subgraph that all the substrates (or products) may include and any conserved partial reactions. A subgroup is essentially created by observing *a similarity* threshold at which all members of the subgroup have more in common with one another than they do with members of another subgroup. (Thresholds derived from similarity calculations can use many different metrics, such as simple database search programs like BLAST [26] or Hidden Markov Models (HMMs) [27] generated as part of the curation protocol to describe a subgroup or family.)

### 1.4 Annotation Transfer and Misannotation

Annotation transfer is a hard problem to solve, partly because it is not always easy to know exactly how a function should be transferred. Oftentimes, function and sequence similarity do not track well [28, 29] and so, if sequence similarity is the only criterion that has been used for annotation transfer, the inference of function may have low confidence. However, it is also very difficult to say whether a protein is truly misannotated, especially if no fairly similar protein has been experimentally characterised that could be used for comparison and evaluation of functional features such as the presence of similar functionally important active site residues. As we have previously shown [30–32] there is a truly staggering amount of protein space that has yet to be explored experimentally and that makes it very difficult to make definitive statements as to the validity of an annotation.

**Fig. 3:**
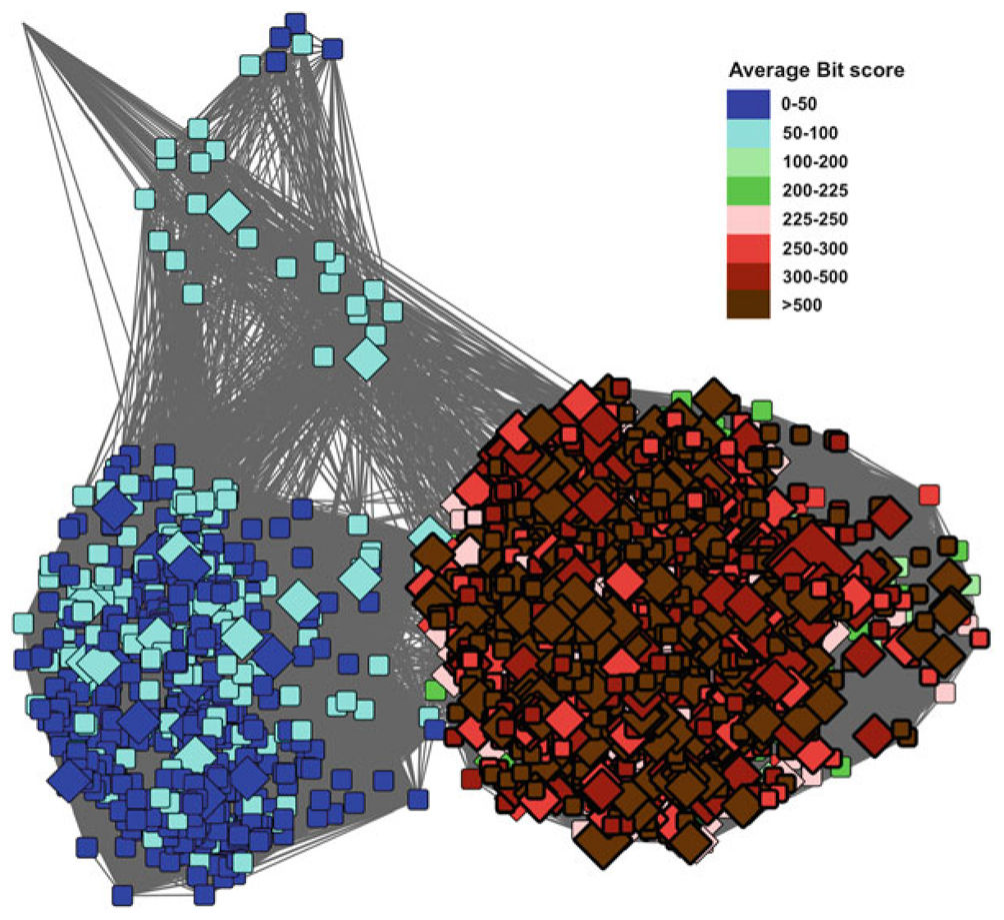
Example of identifying misannotation using an SSN in the biotin synthase- like subgroup in the SFLD. Nodes colours represent different families in the subgroup, where *red* represent those sets of sequences annotated as canonical biotin synthase in the SFLD, *blue* represent the HydE sequences, *green* the PylB sequences and magenta the HmdB sequences. The nodes shown as *large diamonds* are those annotated as BioB in GO, clearly showing that the annotation transfer for BioB is too broad. The network summarizes the similarity relationships between 5907 sequences. It consists of 2547 representative nodes (nodes represent proteins that share greater than 90 % identity) and 2,133,749 edges, where an edge is the average similarity of pairwise BLAST *E*-values between all possible pairs of the sequences within the connected nodes. In this case, edges are included if this average is more significant than an *E*-value of 1e-25. The organic layout in Cytoscape 3.2.1 is used for graphical depiction. Subheading 2.1 described how such similarity networks are created

Misannotation can come from many sources, from a human making an error in curation, which is then propagated from the top down, to an automated annotation transfer rule that is slightly too lax, to the use of transitivity to transfer annotation, e.g. where protein A is annotated with function X, protein B is 70 % identical to A, and so is also assigned function X, protein C is 65 % identical to protein B, and so is also assigned function X. Whilst this may be the correct function, protein C may have a much lower similarity to protein A, and thus the annotation transfer may be “risky” [33]. As in the example shown in Fig. 3, sequence similarity networks (SSNs) [34] offer a powerful way to highlight where potential misannotation may occur. In this network, all the nodes are connected via a homologous domain, the Radical SAM domain. Thus, the observed differences in the rest of the protein mean that the functions of the proteins may also be quite different. For details on the creation of SSNs, *see* Subheading 2.1. Cases where annotations may be suspect can often be evaluated based on a protein’s assigned name, and from the GO terms inferred for that protein.

Not all annotations are created equal, even amongst experimentally validated annotations, and it is important to consider how well evidence supporting an annotation should be trusted. For example, in the glutathione transferase (GST) superfamily, the cognate reaction is often not known as the assays performed use a relatively standard set on non-physiological substrates to infer the type of reaction catalysed by each enzyme that is studied. Moreover, GSTs are often highly promiscuous for two or more different reactions again complicating function assignment [32]. That being said, the availability of even a small amount of experimental evidence can help guide future experiments aimed at functional characterisation. A new ontology, the Confidence Information Ontology (CIO) [16], aims to help annotators assign confidence to evidence. For example, evidence that has been reproduced from many different experiments may have an intrinsically higher confidence than evidence that has only been reported once.

## 2 Using GO Annotations to Visualise Data in Sequence Similarity Networks

**Fig. 4:**
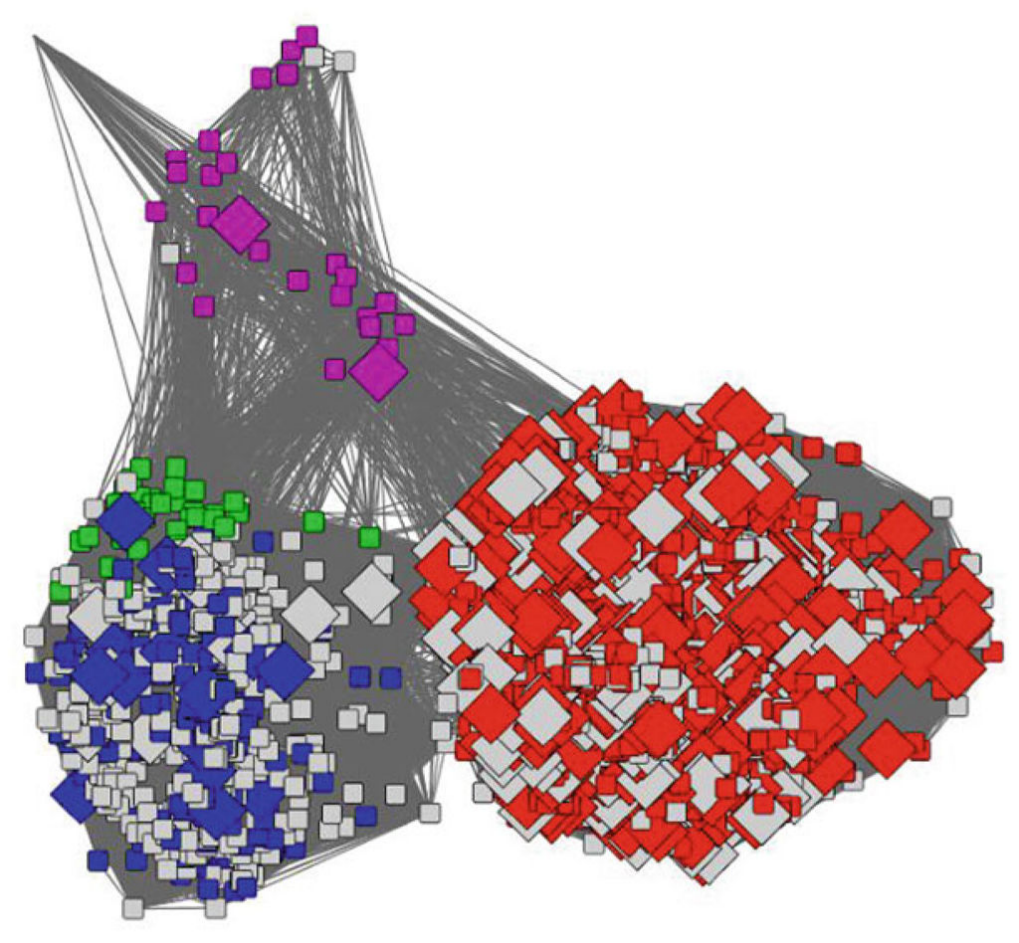
Biotin synthase-like subgroup coloured by confidence of evidence (as shown in Table 1). The *diamond shaped nodes* are all annotated as Biotin Synthase in GO. *Red nodes* are those that only have low confidence annotations, the *orange nodes* are those that have at least one medium-confidence annotations and the *green* are those that have at least one high-confidence annotation. *Grey nodes* have no BioB annotations. Node and edge numbers, as well as *e*-value threshold are as in Fig. 3

Sequence similarity networks (SSNs) are a key tool that we use in the Structure-Function Linkage Database (SFLD) as they give an immediately accessible view of the superfamily and the relationships between proteins in this set. This in turn allows a user to identify boundaries at which they might reasonably expect to see proteins performing a similar function in a similar manner. As was shown in Fig. 3, the GO annotation for BioB covered several different SFLD families. These annotation terms have been assigned through a variety of methods, but mostly inferred from electronic annotation (i.e. rule-based annotation transfer as shown in Fig. 4).

**Fig. 5:**
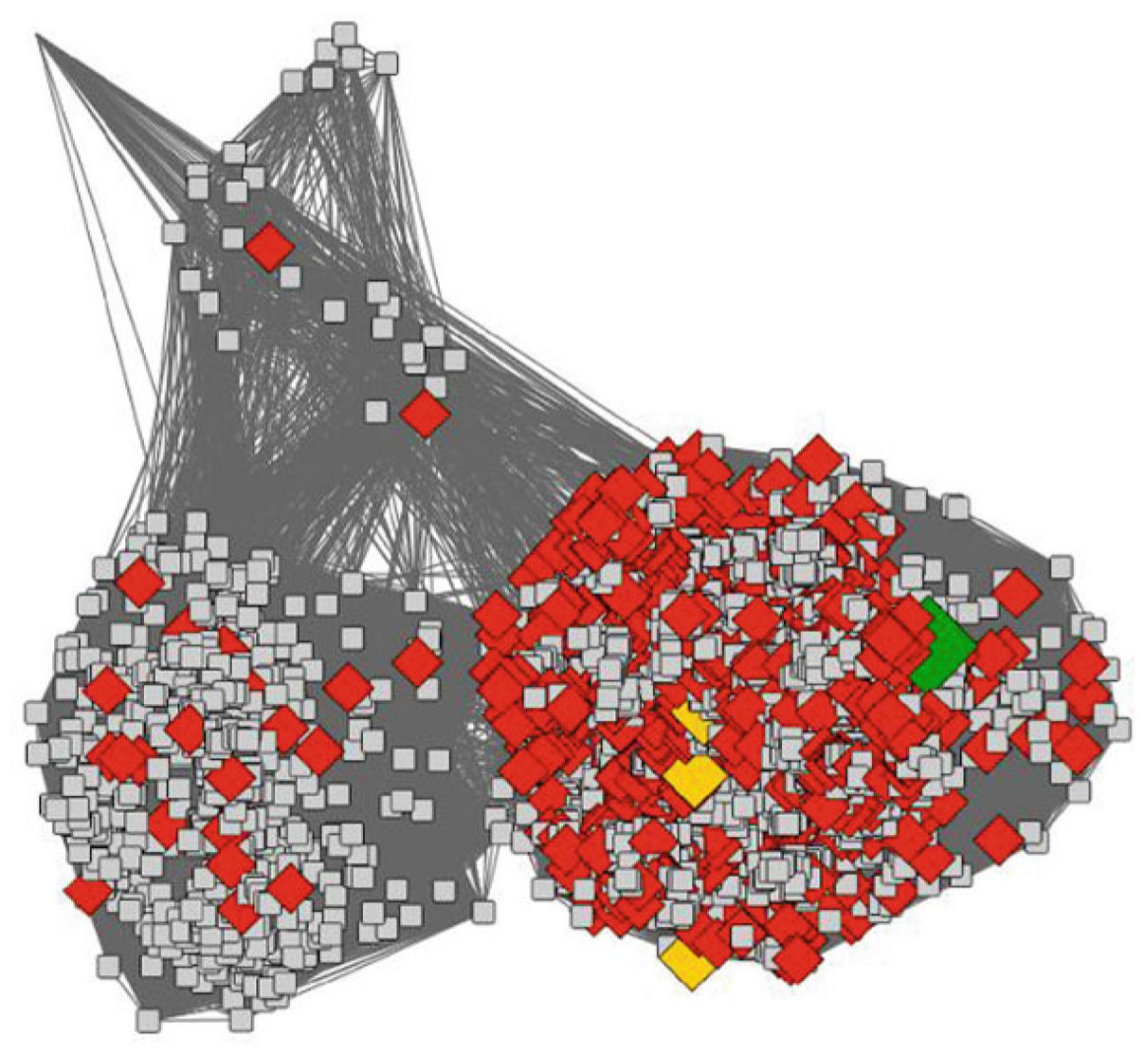
Example of a sequence similarity network to estimate subgroups for use in initial steps of the curation process and to guide fine-tuning the hidden Markov model (HMM) true-positive detection threshold of an enzyme family (here for the Biotin synthase (BioB) family). Node colours represent the average Bit-score of the BioB family HMM for all sequences represented by the node. The mapping between colours and average Bit scores is given in the legend. Nodes with *thick borders* represent proteins that belong to the BioB family according to SFLD annotation. *Diamonds* represent nodes that include proteins with BioB family annotation according to GO. The final BioB HMM detection threshold was achieved for the SFLD by further exploration of more strict *E* -value thresholds for edge representation, and was set to 241.6. Node and edge numbers, as well as *E*-value threshold, are as in Fig. 3

From the networks shown previously, a user may intuitively see that there are three basic groups of proteins. Further, it could be hypothesised that these groups could have different functions (which is indeed the case in this particular example). Thus, the user may be left with the question: How do I know what boundaries to use for high confidence in the annotation transfer? Figure 5 shows another network, this time coloured by the average bit-score for the sequences in a node against the SFLD HMM for BioB. This network exemplifies how (1) sequence similarity (network clusters) corresponds with the sequence pattern generated by SFLD curators to represent the BioB family, and (2) HMM true-positive gathering bit-score cut-off can be fine-tuned. By combining what we know about the protein set from the GO annotation (Fig. 3) with the HMM bit-score (Fig. 5) it is possible to be much more confident in the annotations for the proteins in the red/brown group in Fig. 5.

### 2.1 Creating Sequence Similarity Networks

SSNs provide a visually intuitive method for viewing large sets of similarities between proteins [34]. Although their generation is subject to size limitations for truly large data sets, they can be easily created and visualised for several thousand sequences. There are many ways to create such networks, the networks created by the SFLD are generated by Pythoscape [35], a freely available software that can be downloaded, installed and can be run locally. Recently, web servers have been described that will generate networks for users. For example, The Enzyme Similarity Tool (EFI-EST) [36] created by the Enzyme Function Initiative will take a known set of proteins (e.g. Pfam or InterPro [37] groups) and generate networks for users from that set. A similarity network is simply a set of nodes (representing a set of amino acid sequences as described in this chapter, for example) and edges (representing the similarity between those nodes). For the SSNs shown in this chapter, edges represent similarities scored by pairwise BLAST *E*-values (used as scores) between the source and target sequences. Using simple metrics such as these, relatively small networks are trivial and fast to produce from a simple all-against-all BLAST calculation. However, the number of edges produced depends on the similarity between all the nodes to each other, so that for comparisons of a large number of closely related sequences, the number of edges will vastly exceed the number of nodes, quickly outpacing computational resources for generating and viewing networks. As a result, some data reduction will eventually be necessary. The SFLD uses representative networks where each node represents a set of highly similar sequences and the edges between them represent the mean *E*-value similarity between all the sequences in the source node and all the sequences in the target node. As shown in Fig. 3, node graphical attributes (e.g. shape and colour) used to represent GO terms for the proteins shown are a powerful way to recognise relationships between sequence and functional similarities. Importantly, statistical analyses must be carried out to verify the significance of these trends, as we show below.

### 2.2 Determining Over- and Under- represented GO Terms in a Set of Species- Diverse Proteins

A common use of GO enrichment analysis is to evaluate sets of differentially expressed genes that are up- or down-regulated under certain conditions [38]. The resulting analysis identifies which GO terms are over- or under-represented within the set in question. With respect to enzyme superfamilies, the traditional implementation of enrichment analysis will not work well as there are often very many different species from different kingdoms in the dataset. However, there are several ways that we can still utilise sets of annotated proteins to evaluate the level of enrichment for GO terms.

The simplest method and least rigorous, is to take the set of proteins being evaluated, count up the number of times a single annotation occurs (including duplicate occurrences for a single enzyme, as these have different evidence sources) and up-weight for experimental (or high confidence) annotations. Then, by dividing by the number of proteins in the set, any annotation with a ratio greater than one can be considered “significant”.

A more rigorous treatment assumes that for a set of closely related proteins (i.e. belonging to a family) a specific GO term is said to be over-represented when the number of proteins assigned to that term within the family of interest is enriched versus the background model as determined by a probability distribution. Thus, there are two decisions that need to be made, firstly, identifying the background model and then which probability function to use. The background model is dependent on the dataset and the question that is being asked. For example in the SFLD model, we might use the subgroup or superfamily and a random background model that gives us an idea of what annotations could occur purely by chance. The lack of high (and sometimes also medium) confidence annotations is another complication in examining enrichment of terms. If one is using IEA annotations to infer function, the assertions can quickly become circular (with inferred annotations being transferred to other proteins which in turn are used to annotate yet more proteins), leading to results which themselves are of low confidence. Similarly, if very few proteins are explicitly annotated with a high/medium confidence annotation, the measure of significance can be skewed due to low counts in the dataset. The choice of the probability function is also going to depend somewhat on what question is being asked, but the hypergeometric test (used for a finite universe) is common in GO analyses [39, 40]. For more detail on enrichment analysis, *see* Chap. 13 [41].

### 2.3 Using Semantic Significance with GO

Instead of simply transferring annotations utilising sequence homology and BLAST scores, many tools are now available (e.g. Argot2 [42] and GraSM [43]) that utilise semantic similarity [42–46]. Here, the idea is that in controlled vocabularies, the degree of relatedness between two entities can be assessed by comparing the semantic relationship (meanings) between their annotations. The semantic similarity measure is returned as a numerical value that quantifies the relationship between two GO terms, or two sets of terms annotating two proteins.

GO is well suited to such an approach, for example many children terms in the GO directed acyclic graph (DAG) have a similar vocabulary to their parents. The nature of the GO DAG means that a protein with a function A will also inherit the more generic functions that appear higher up in the DAG; this can be one or more functions, depending on the DAG. For example, an ion transmembrane transporter activity (GO:0015075) is a term similar to voltage-gated ion channel activity (GO:0005244), the latter of which is a descendent of the former, albeit separated by the ion channel activity (GO:0005216) term. Thus, the ancestry and semantic similarity lends greater weight to the confidence in the annotation.

Such similarity measures can be used instead of (or in conjunction with) sequence similarity measures. Indeed, it has been shown [47] that there is good correlation between the protein sequence similarity and the GO annotation semantic similarity for proteins in Swiss-Prot, the reviewed section of UniProtKB [17]. Consistent results, however, are often a feature not only of the branch of GO to which the annotations belong, but also the number of high confidence annotations that are being used. For a more detailed and comprehensive discussion of the various methods, *see* Pesquita et al. [44] and Chap. 12 [48].

### 2.4 Use of Orthogonal Information to Evaluate GO Annotation

In the example shown in Fig. 3, it is clear that many more nodes in the subgroup are annotated as biotin synthase by GO than match the stringent criteria set within the SFLD, which not only require a significant *E*-value (or Bit Score) to transfer annotation, but the presence of the conserved key residues. As mentioned earlier, one key advantage to using GO annotations over those of some other resources is the evidence code (and associated source of that evidence) as shown in Fig. 4. As indicated by that network, when using GO annotations, it is important to also consider the associated confidence level for the evidence used in assigning an annotation (*see* Table 1). In Fig. 4, only a few annotations are supported by high-confidence evidence. Alternatively, if a protein has a high confidence experimental evidence code for membership in a family of interest yet is not included by annotators in that family, then the definition of that family may be too strict, indicating that a more permissive gathering threshold for assignment to the family should be used.

Another way of assessing the veracity of the annotation transferred to a query protein is to examine both the annotations of the proteins that are closest to it in similarity as well as other entirely different types of information.

One example of such orthogonal information is the genomic context of the protein. It can be hypothesised that if a protein occurs in a pathway, then the other proteins involved in that pathway may be co-located within the genome [49]. This association is frequently found in prokaryotes, and to a lesser extent in plants and fungi. Genomic proximity of pathway components is infrequent in metazoans, thus genomic context as a means to function prediction is more useful for bacterial enzymes. Additionally, other genes in the same genomic neighbourhood may be relevant to understanding the function of both the protein of interest and of the associated pathway. A common genomic context for a query protein and a homologue provides further support for assignment of that function. (However, the genomic distance between pathway components in different organisms may vary for many reasons, thus the lack of similar genomic context does not suggest that the functions of a query and a similar homologue are different.)

**Fig. 6:**
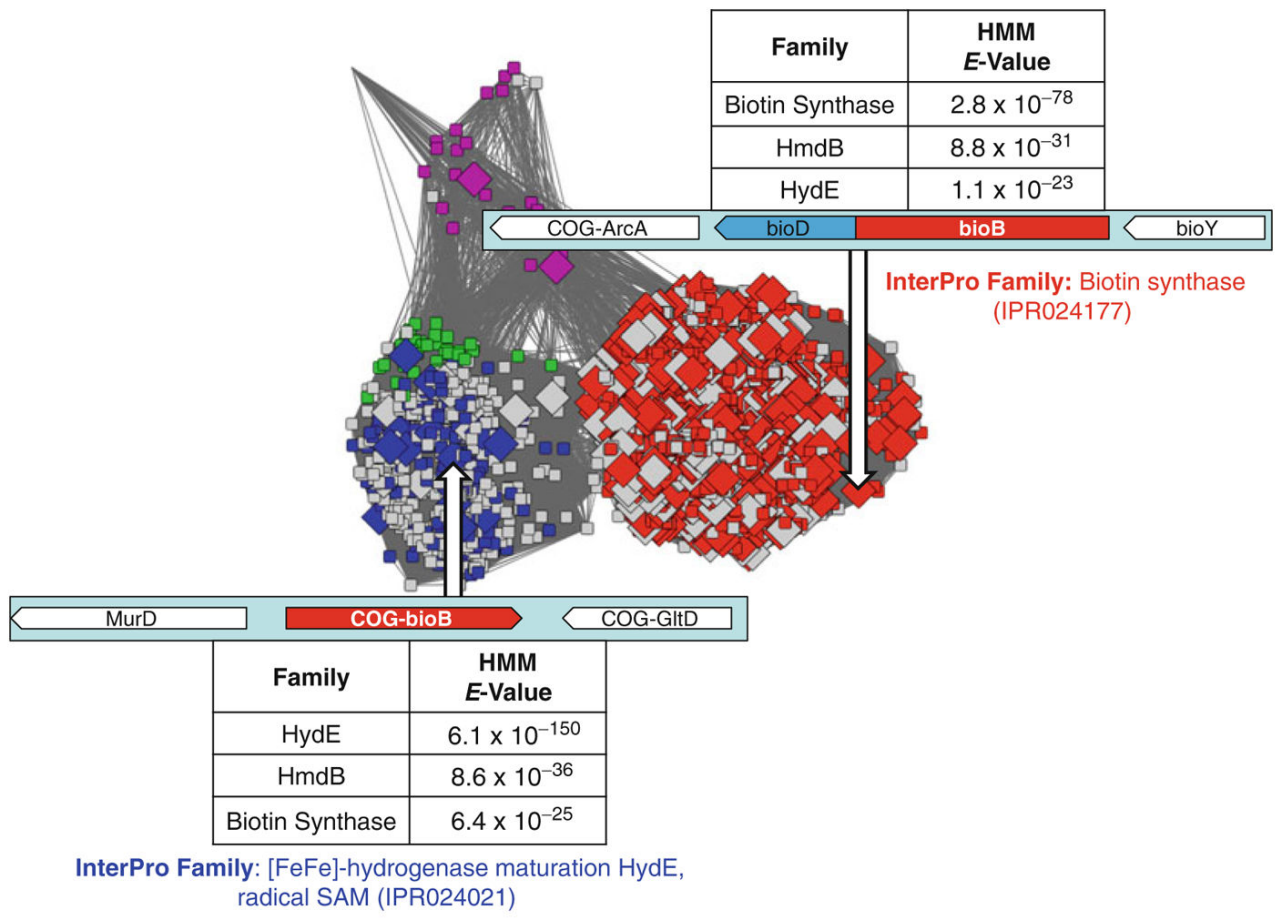
Biotin synthase-like subgroup SSN showing where the biotin synthase GO annotations are shown as *large diamonds* . Two proteins, one from the BioB set (*red nodes, top right* ) and one from the HydE set (*blue nodes*), *bottom left* , are shown with some associate orthogonal information: genomic context highlighted in *light cyan boxes*, their HMM match results for the query protein against the three top scoring families in the subgroup are shown in the tables, and family membership (according to InterProScan) shown in coloured text (*blue* for HydE and *red* for BioB). Node and edge numbers, as well as *E*-value threshold are as in Fig. 3. All the proteins are connected via a homologous domain (the Radical SAM domain). Thus, the observed differences in the rest of the protein mean that the functions of the proteins may also be quite different

Another type of orthogonal information that can be used can be deduced from protein domains present in a query protein and their associated annotations—what are the predicted domains present in the protein, do they all match the assigned function or are there anomalies. A good service for identifying such domains is InterProScan [50]. Further, any protein in UniProtKB will have the predicted InterPro identifiers annotated in the record (along with other predicted annotations from resources such as Pfam and CATH), along with the evidence supporting those predictions. Such sequence context can also be obtained using hidden Markov models (HMMs) [51], which is the technique used by InterPro, Pfam, Gene3D, SUPERFAMILY and the SFLD to place new sequences into families, subgroups (SFLD-specific term) and superfamilies (*see* Fig. 6).

## 3 Challenges and Caveats

### 3.1 The Use of Sequence Similarity Network

A significant challenge with using SSNs to help evaluate GO annotations is that SSNs are not always trivial to use without a detailed knowledge of the superfamilies that they describe. For example, choosing an appropriate threshold for drawing edges is critical to obtaining network clustering patterns useful for deeper evaluation. In Fig. 3, HydE (the blue nodes) are not currently annotated as such in GO, but are annotated instead as BioB. Thus, the evaluation of the network becomes significantly more complex. It is also not always clear what signal is being picked up in the edge data for large networks. It is usually assumed that all the proteins in the set share a single domain, but this is often only clear when the network is examined in greater detail.

### 3.2 Annotation Transfer Is Challenging Because Evolution Is Complex

Even using the powerful tools and classifications provided by GO, interpreting protein function in many cases requires more in-depth analysis. For several reasons, it is not always easy to confidently determine that a protein is not correctly annotated. Firstly, how closely related is the enzyme to the group of interest? Perhaps we can only be relatively certain of its superfamily membership, or maybe we can assign it to a more detailed level of the functional hierarchy. If it fits into a more detailed classification level, how well does it fit? At what threshold do we begin to see false positives creeping into the results list? Using networks, we can also examine the closest neighbours that have differing function and ask whether there are similarities in the function (e.g. Broderick et al. [52] used sequence similarity networks to help determine the function of HydE). Another complicating issue is whether a protein performs one or more promiscuous functions, albeit with a lesser efficacy.

Another important piece of evidence that can be used to support an annotation is conservation of the key residues, so it is important to assess if the protein of interest has all the relevant functional residues. Although GO includes an evidence code to handle this concept (Inferred from Key Residues, IKR), it is often not included in the electronic inference of annotations. It is important to note, however, that there are evolutionary events that may “scramble” the sequence, leaving it unclear to an initial examination whether the residues are conserved or not. A prime example is the case in which a circular permutation has occurred. Thus, it is important to look at whether there are other residues (or patterns of residues) that could perform the function of the “missing” residues. It is also possible that conservative mutations have occurred, and these may also have the ability to perform the function of the “missing” residues [53].

Another consideration with function evaluation is the occurrence of moonlighting proteins. These are proteins that are identical in terms of sequence but perform different functions in different cellular locations or species; for example argininosuccinate lyase (UniProtKB id P24058) is also a delta crystalline which serves as an eye lens protein when it is found in birds and reptiles [54]. A good source of information on moonlighting proteins is MoonProt (http://www.moonlightingproteins.org/) [55]. Such cases may arise from physiological use in many different conditions such as different subcellular localisations or regulatory pathways. The full extent of proteins that moonlight is currently not known, although to date, almost 300 cases have been reported in MoonProt. Another complicating factor for understanding the evolution of enzyme function is the apparent evolution of the same reaction specificity from different intermediate nodes in the phylogenetic tree for the superfamily, for example the N-succinyl amino acid racemase and the muconate lactonising enzyme families in the enolase superfamily [56, 57].

Finally, does the protein have a multi-domain architecture and/ or is it part of a non-covalent protein-protein interaction in the cell? An example of a functional protein requiring multiple chains that are transiently coordinated in the cell is pyruvate dehydrogenase (acetyl-transferring) (EC 1.2.4.1). This protein has an active site at the interface between pyruvate dehydrogenase E1 component subunit alpha (UniProtKB identifier P21873) and beta (UniProtKB identifier P21874), both of which are required for activity. Thus, transfer of annotation relating to this function to an unknown (and hence evaluation of misannotation) needs to include both proteins. Similarly, a single chain with multiple domains, e.g. biotin biosynthesis bifunctional protein BioAB (UniProtKB identifier P53656), which contains a BioA and BioB domain, has two different functions associated with it. In this example, these two functions are distinct from one another so that annotation of this protein only with one function or the other could represent a type of misannotation (especially as a GO term is assigned to a protein, not a specific segment of its amino acid sequence).

### 3.3 Plurality Vote May Not Be the Best Route

In some cases, proteins are annotated by some type of “plurality voting”. Plurality voting is simply assuming that the more annotations that come from different predictors, the more likely these are to be correct. As we have shown in this chapter (and others before us [58]), this is not always the case. An especially good example of where plurality voting fails is in the case of the lysozyme mechanism. For over 50 years, the mechanism was assumed to be dissociative, but a single experiment provided evidence of a covalent intermediate being formed in the crystal structure, calling into question the dissociate mechanism. If plurality voting were applied in ongoing annotations, the old mechanism would still be considered correct. That being said, it is more difficult to identify problems of this type if experimental evidence challenging an annotation is unavailable. In such cases, we must always look at all the available evidence to transfer function and where there are disagreements between predicted functions, a more detailed examination is needed. Only when we have resolved such issues can we have any true confidence in the plurality vote. Work by Kristensen et al. [59] provides a good example of the value of this approach. By using three-dimensional templates generated using knowledge of the evolutionarily important residues, they showed that they could identify a single most likely function in 61 % of 3D structures from the Structural Genomics Initiative, and in those cases the correct function was identified with an 87 % accuracy.

## 4 Conclusions

Experimentalists simply can’t keep up with the huge volume of data that is being produced in today’s high-throughput labs, from whole genome and population sequencing efforts to large-scale assays and structure generation. Almost all proteins will have at least one associated GO annotation, and such coverage makes GO an incredibly powerful tool, especially as it has the ability to handle all the known function information at different levels of biological granularity, has explicit tools to capture high-throughput experimental data and utilises an ontology to store the annotation and associated relationships. Although over 98 % of all GO annotations are computationally inferred, with the ever-increasing state of knowledge, these annotation transfers are becoming more confident [15] as rule-based annotations gain in specificity due to more data being available. However, there is still a long way to go before we can simply take an IEA annotation at face value. Confidence in annotations transferred electronically has to be taken into account: How many different sources have come to the same conclusion (using different methods)? How many different proteins’ functions have been determined in a single experiment? Similarly, whilst burden of evidence is a useful gauge in determining the significance of an annotation, there is also the question of when substantially different annotations were captured in GO and other resources—perhaps there has been a new experiment that calls into question the original annotation. It is also important to look at whether other, similar proteins were annotated long ago or are based on new experimental evidence. There is a wealth of data available that relates to enzymes and their functions. This ranges from the highest level of associating a protein with a superfamily (and thus giving some information as to the amino acid residues that are evolutionarily conserved), to the most detailed level of molecular function. We can use all of these data to aid us in evaluating the GO annotations for a given protein (or set of proteins), from the electronically inferred annotation for protein domain structure, to the genomic context and protein features (such as conserved residues). The more data that are available to back up (or refute) a given GO annotation, the more confident one can be in it (or not, as the case may be).

## Figures and Tables

**Table 1:** Some example proteins (listed by UniProtKB accession) with their associated annotations, source of the annotation (the SFLD is the Structure-Function Linkage Database, Swiss-Prot is the curated portion of UniProtKB) and the confidence of those annotations along with the reason that confidence level has been assigned

Protein ID from UniProtKB [17]	Annotated protein function (*source*)	SFLD confidence level	Types of evidence or reasoning used to annotate the function
Q9X0Z6	[FeFe]-hydrogenase maturase (*From SFLD and Swiss-Prot*)	High	Inferred from experimental analysis of protein structures, genomic context and results from spectroscopic assay.
Q11S94	Biotin Synthase (BioB) (*From SFLD and Swiss-Prot*)	Medium	Inferred from similarity to other BioB enzymes. Matched by similarity to other BioB sequences and catalytic residues are fully conserved.
Q58692	Biotin Synthase (BioB) (*From Swiss-Prot*)	Low	Inferred from similarity to other BioB enzymes. Matched by similarity to other BioB sequences. Whilst all residues required for binding the iron-sulphur clusters are conserved, all the catalytic residues (those required for the BioB reaction to occur) are not. Also has no biotin synthase genomic context.
